# Vascularized bone graft and scapholunate fixation for proximal scaphoid nonunion: a case report

**DOI:** 10.1080/23320885.2020.1791715

**Published:** 2020-07-13

**Authors:** Shu-Hsin Yao, Jung-Pan Wang, Hui-Kuang Huang

**Affiliations:** a Department of Orthopaedics, Chiayi Christian Hospital, Chiayi, Taiwan; b Department of Surgery, School of Medicine, National Yang-Ming University, Taipei, Taiwan; c Department of Orthopaedics & Traumatology, Taipei Veterans General Hospital, Taipei, Taiwan; d Chung Hwa University of Medical Technology, Tainan, Taiwan

**Keywords:** 23 ICSRA, nonunion, proximal scaphoid, scapholunate, vascularized bone graft

## Abstract

We report a 38-year-old woman with a proximal scaphoid fracture nonunion from an injury 1 year ago. She was successfully treated with 2,3 intercompartmental supraretinacular artery pedicled vascularized bone graft and scapholunate fixation. It highlights the possible combination of scapholunate fixation and vascularized bone grafting in treating proximal scaphoid nonunion.

## Introduction

Treatment for proximal scaphoid nonunion can be challenging, since there could be some difficulties, including poor proximal fragment vascularity, possible scapholunate (SL) interosseous ligament injury, and limited space for stable hardware fixation [1,2].

For scaphoid nonunion with ischemic change, pedicled vascularized bone graft (VBG) or free VBG could be an option to enhance circulation and facilitate healing [3]. For fixation of a proximal scaphoid fracture, no matter whether K-wires or screws are used, some surgeons would feel that fixation to a limited space in the proximal scaphoid fragment is a challenge. Fixation from the scaphoid to the lunate in treating proximal scaphoid nonunion has been reported by Slade and Dodds to achieve a more stable fixation [4]. This is because the small proximal scaphoid fragment could be fixed sandwich-like between the distal scaphoid fragment and the lunate.

Here, we report a case of proximal scaphoid nonunion and ischemic change of the scaphoid. The patient was treated with 2,3 intercompartmental supraretinacular artery (ICSRA) VBG and SL screw fixation.

## Case report

**Figure 1.**
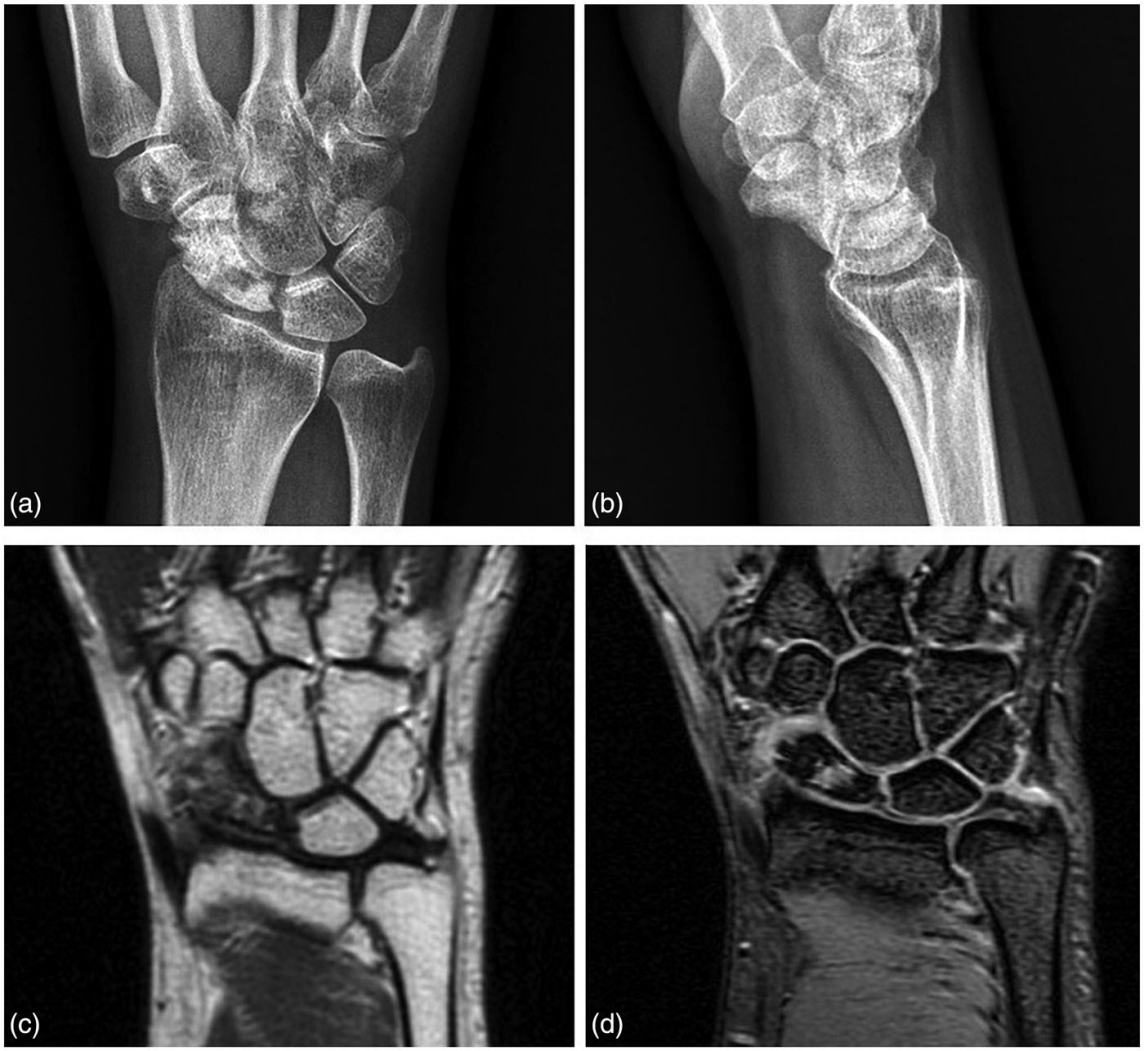
(a,b) Radiographs showing the right proximal scaphoid fracture nonunion with bony density change; (c) Magnetic resonance imaging shows ischemic change with a diffuse hypointense marrow signal in the scaphoid fragments and (d) a nonunion cavity.

A 38-year-old right-handed woman experienced an accidental fall more than 1 year before she presented to our clinic. She initially went to an orthopedic clinic for evaluation. She was told she had wrist contusion only and was treated for wrist pain. At our clinic, she complained of persistent pain and limited range of motion (ROM) in her right wrist. The measurement of her wrist motion was flexion 30° and extension 10°. Radiographs showed proximal scaphoid fracture nonunion with scaphoid density change, but no obvious humpback deformity. MRI also showed scaphoid ischemic change with a nonunion cavity (Figure 1). We then decided to use VBG to treat the fracture nonunion. SL screw fixation was applied as the proximal scaphoid fragment was small and the fixation stability for both the fracture and VBG could not be ensured.

**Figure 2.**
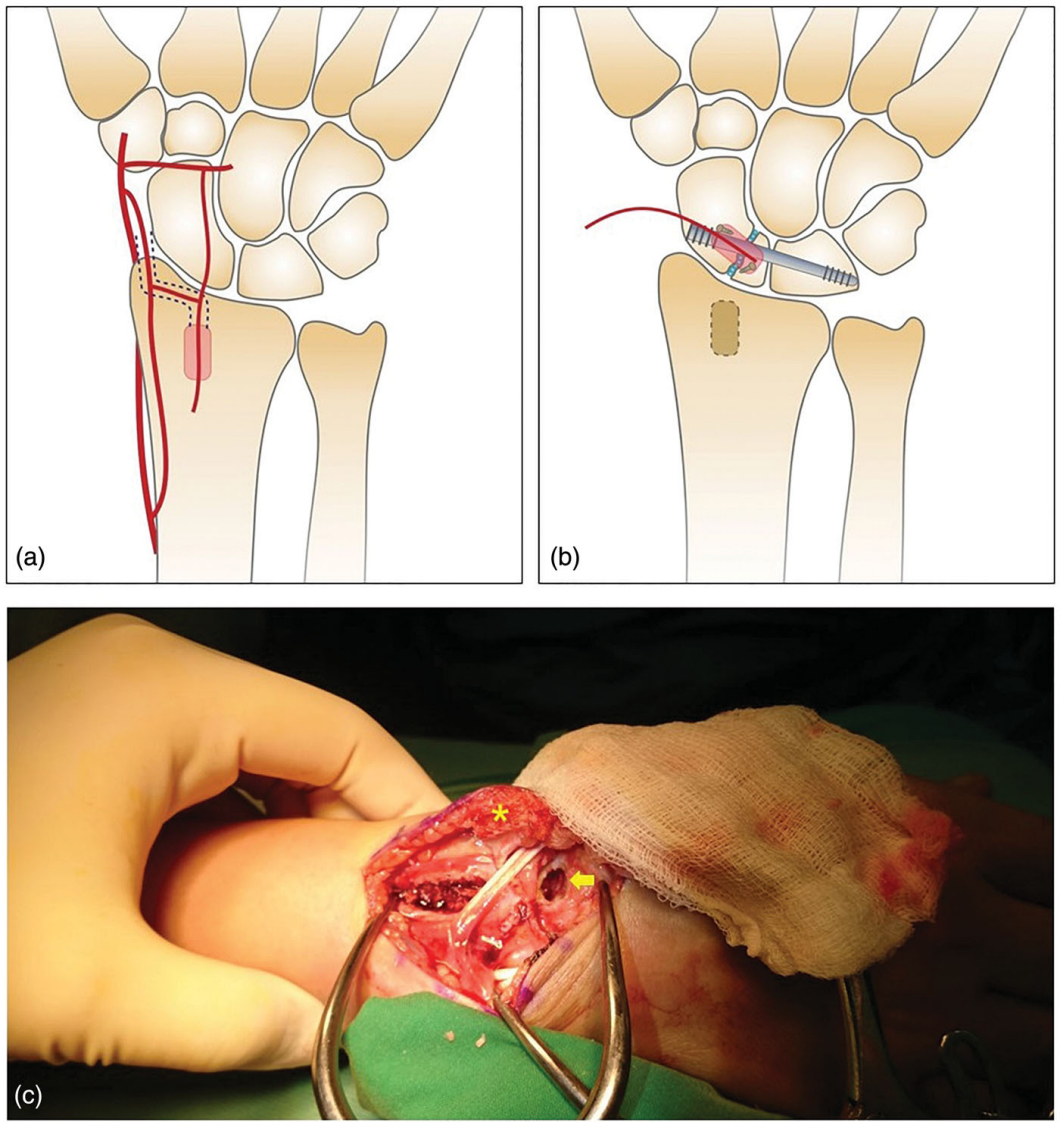
Schematic diagram of (a) the 2,3 ICSRA pedicled vascularized bone graft designed for harvesting and (b) fixation of the scapholunate screw and vascularized bone graft. (c) Photograph depicting the harvested 2,3 ICSRA pedicled vascularized bone graft (asterisk) and the created cavity on the scaphoid (arrow) for setting of the vascularized bone graft.

2,3 ICSRA VBG was harvested first from a longitudinal dorsal wrist incision, based on Woon Tan and Tu’s method [5] (Figure 2). Then, with a transverse capsulotomy, debridement of the necrotic bone of the nonunion site was performed until vital bone was seen. Bleeding from the debrided scaphoid bone edges through deflation of the arm tourniquet could help ensure an adequate debridement. Additional cancellous bone graft was harvested from the VBG donor site to fill the debrided defect, which was up to 8 mm, in order to maintain the scaphoid length and avoid the bridging defect.

**Figure 3.**
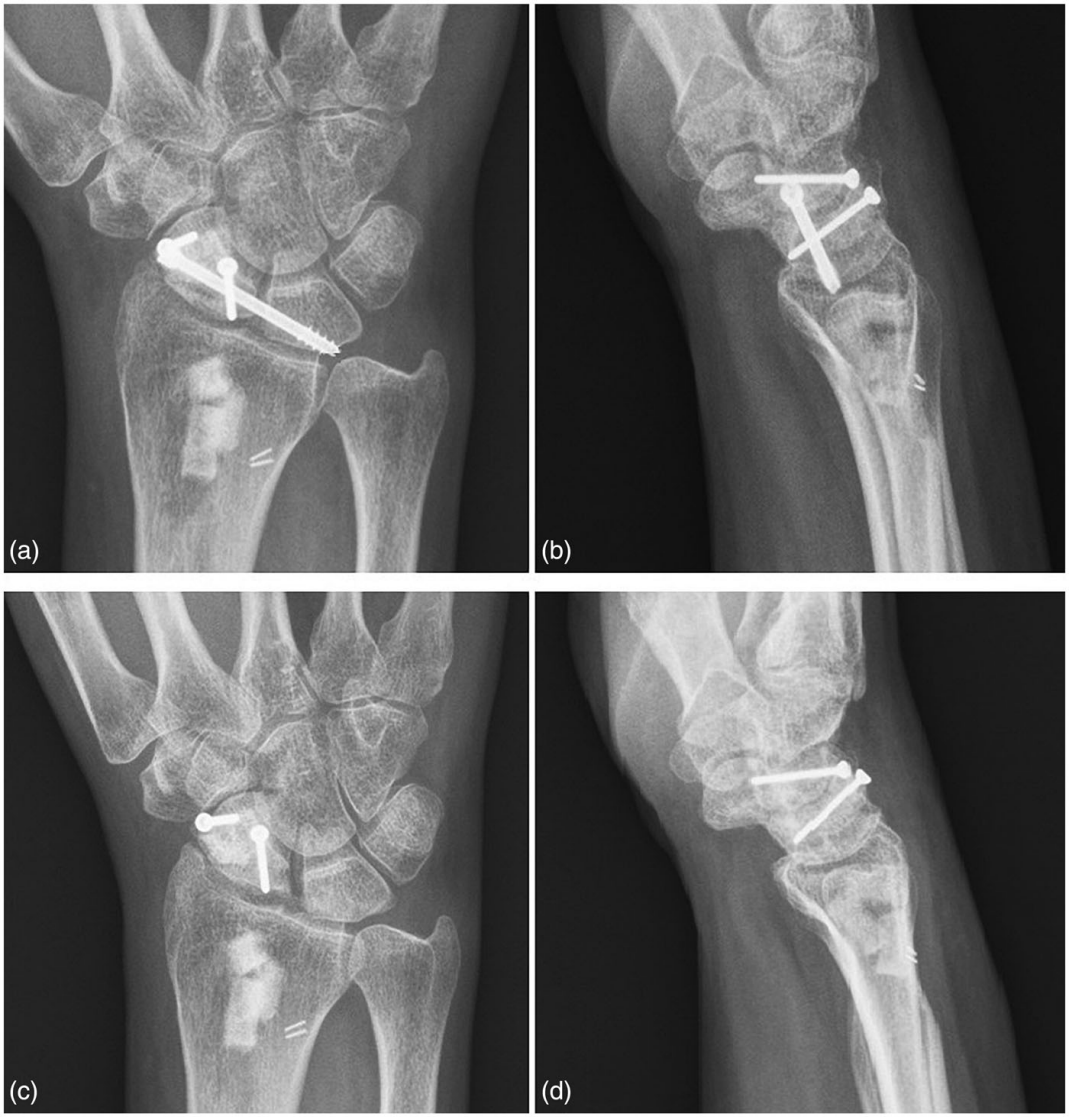
(a,b) Radiographs showing the right scaphoid after vascularized bone grafting and scapholunate screw fixation at postoperative 3 months. (c,d) Radiographs at postoperative 2 years reveal consolidated union (the screw had been removed 6 months after surgery because it was too long to penetrate the proximal cortex of the lunate).

Under fluoroscopy, we set a 3.0-mm headless compression screw (DePuy Synthes, West Chester, PA) for SL fixation with a small incision at the anatomical snuffbox for vital structures protection [4]. Then, we used a low-speed burr and curette to create a cavity centered on the fracture and sitting on both poles of the scaphoid for fitting of the VBG. The VBG was set into the created cavity and secured with 2 1.5-mm mini-screws on the distal and proximal fragments separately (Figures 2 and 3). It would be better to perform SL screw fixation prior to setting the VBG on the scaphoid, because setting the mini-screws for securing the VBG would be easier at that time, as their directions would need to be adjusted so as not to hit on the headless screw.

After surgery, a short-arm plaster thumb spica splint was used for 1 month. Then, a removable wrist brace was used until fracture healing was noted. Gentle wrist ROM exercise was initiated 2 months after surgery. Strengthening exercises for the wrist were started when the radiographic and clinical fracture healing was seen.

At the 2-year follow-up, her wrist flexion had improved from 30° to 55°, extension from 10° to 45°, and grip strength from 10 kg to 18.9 kg. And at the final follow-up, the visual analog pain score (0, no pain; 10, worst pain) was 0 at rest and 2 during activities.

## Discussion

Whether vascularized or non-vascularized bone graft is needed for a scaphoid nonunion site is still a matter of debate [6]. Many authors have reported that after a detailed debridement of the nonunion site, the use of non-vascularized bone graft would be enough for fracture healing of the proximal scaphoid nonunion, even though there is evidence of ischemic change [7–9]. A VBG could be used to optimize revascularization and fracture-healing biology in the presence of avascular necrosis or previous non-vascularized bone graft failure [3].

A headless screw for intrascaphoid fixation, either antegrade or retrograde, could be used for proximal scaphoid fracture fixation. Although there are no significant biomechanical strength differences between the 2 screw configurations for fixation of the proximal pole [10], the limited space in the proximal scaphoid fragment for screw insertion and securing would be considered a difficulty by some surgeons. When VBG is used for proximal scaphoid nonunion treatment, especially, the level of difficulty would increase if the screw is planned to be well secured without hindering the VBG setting.

With SL fixation, the screw would have more bony purchase in the lunate, which could provide better stability for the proximal scaphoid fragment than that with screw fixation in the scaphoid only. Another benefit would be that if there is an SL injury concomitant with the scaphoid fracture, even though most concomitant ligamentous injuries would be mild, screw transfixation for the SL interval would be a possible treatment [11].

The use of K-wires for fixation would have been a good choice in this case. They would be easy to remove and the small diameter of the K-wires would lead to fewer complications than other headless compression screw fixations. But our only concern with the use of K-wires was that the healing time for such a sclerotic scaphoid could not be predicted precisely, so K-wires-related skin irritation and the inconvenience to the patient’s daily life and self-care would be a problem for an indefinite period.

Arthroscopic debridement and bone grafting is another method to treat scaphoid nonunion, even when there is ischemic change, and the results have been reported to be good [12]. With the more limited violation of the surrounding soft tissue, it would be possible to maintain more circulation to the scaphoid. Furthermore, the minimized invasiveness of the surgery would likely cause less scar tissue formation. All of these factors would be beneficial for fracture healing and wrist motion recovery. So, in cases such as this, arthroscopic bone grafting and the use of SL fixation may also be a choice of treatment if the surgeons were familiar with arthroscopic treatment.

For this patient with a proximal scaphoid nonunion with avascular change, scaphoid volar plate fixation with autogenous cancellous bone grafting could be a good choice, and has been reported to be successful [13,14]. With a volar approach to the scaphoid nonunion, the deformity of the scaphoid nonunion would be easier to address. The precontoured scaphoid plate would also be helpful for scaphoid reduction. In addition, the proximal small fragment could be fixed stably with small screws. This could minimize the space-occupying metal within the bone and maximize the bony contact surface for healing. However, the scaphoid plate is not available in our institute.

There are some pitfalls and pearls in the use of this method for treatment of proximal scaphoid nonunion. If the proximal scaphoid fragment is too small, we do not suggest to use this method. Because a large headless screw combined with a mini-screw will take up too much bone stock of the proximal pole. Also, the headless screw should be inserted meticulously to avoid pushing away the proximal fragment. It is important to advance the headless screw under fluoroscopy while engaging the proximal fragment. As to the screw length, we would suggest to subtract 4 mm off the measured length, just like that which is suggested in the method of reduction association of the scapholunate joint [15]. In this case, only 2 mm subtracting off the measured length would lead to a too long screw so that hardware removal should be planned (Figures 3). In addition, CT imaging would be helpful in confirming the bony healing, although we did not obtain it, with which the strengthening exercises could be started promptly.

In this case, with proximal scaphoid fracture nonunion with scaphoid ischemic change, we found that VBG could be combined with SL fixation. The proximal scaphoid fragment could be secured well, and VBG setting would become easier. Also, fracture healing and satisfactory functional outcomes could be achieved.
